# 2-Phenyl­ethanaminium 4-hy­droxy­benzoate

**DOI:** 10.1107/S1600536813010787

**Published:** 2013-04-27

**Authors:** S. Sudhahar, M. Krishnakumar, B. M. Sornamurthy, G. Chakkaravarthi, R. Mohankumar

**Affiliations:** a Department of Physics, Presidency College, Chennai 600 005, India; b Department of Physics, CPCL Polytechnic College, Chennai 600 068, India

## Abstract

In the title salt, C_8_H_12_N^+^·C_7_H_5_O_3_^−^, the cation is disordered over two orientations with site occupancies of 0.565 (7) and 0.435 (7). In the anion, the carboxyl­ate group makes the dihedral angle of 4.19 (18)° with the benzene ring. In the crystal, the ions are connected by N—H⋯O and O—H⋯O hydrogen bonds, forming a three-dimensional network.

## Related literature

For structures containing *p*-hy­droxy­benzoate anions, see: Marsh & Spek (2001 ▶); Yang *et al.* (2010 ▶); Sudhahar *et al.* (2013 ▶).
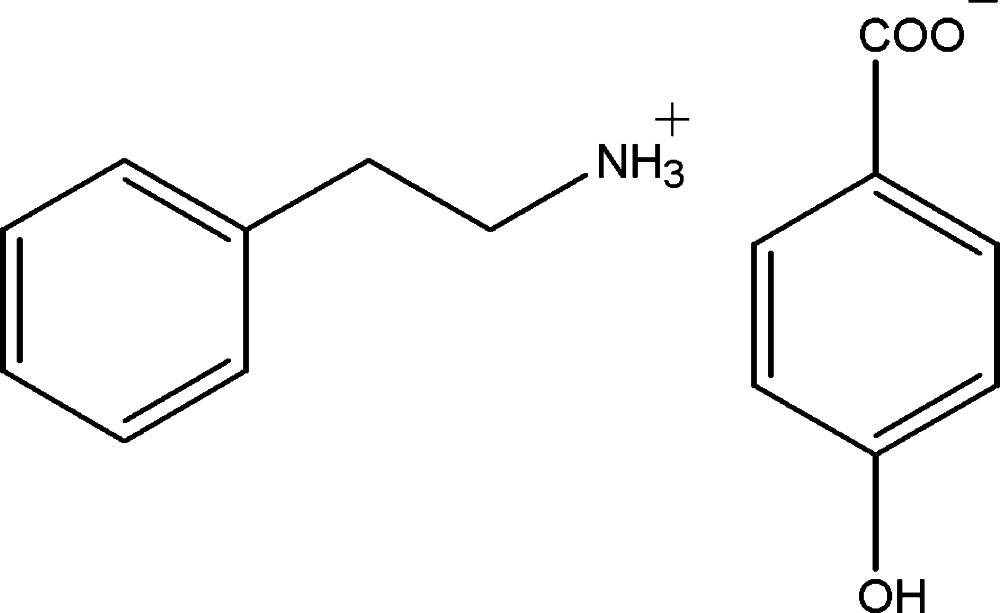


## Experimental

### 

#### Crystal data

C_8_H_12_N^+^·C_7_H_5_O_3_^−^
*M* *_r_* = 259.30
Orthorhombic, 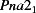

*a* = 13.0721 (12) Å
*b* = 17.3426 (16) Å
*c* = 6.2154 (6) Å
*V* = 1409.1 (2) Å^3^
*Z* = 4
Mo *K*α radiation
μ = 0.09 mm^−1^
*T* = 295 K
0.36 × 0.30 × 0.24 mm

#### Data collection

Bruker Kappa APEXII CCD diffractometer
Absorption correction: multi-scan (*SADABS*; Sheldrick, 1996 ▶) *T*_min_ = 0.970, *T*_max_ = 0.980
7327 measured reflections
2561 independent reflections
1671 reflections with *I* > 2σ(*I*)
*R*_int_ = 0.029

#### Refinement

*R*[*F* ^2^ > 2σ(*F* ^2^)] = 0.038
*wR*(*F* ^2^) = 0.106
*S* = 1.03
2561 reflections
254 parameters
6 restraints
H-atom parameters constrained
Δρ_max_ = 0.12 e Å^−3^
Δρ_min_ = −0.13 e Å^−3^

### 

Data collection: *APEX2* (Bruker, 2004 ▶); cell refinement: *SAINT* (Bruker, 2004 ▶); data reduction: *SAINT*; program(s) used to solve structure: *SHELXS97* (Sheldrick, 2008 ▶); program(s) used to refine structure: *SHELXL97* (Sheldrick, 2008 ▶); molecular graphics: *PLATON* (Spek, 2009 ▶); software used to prepare material for publication: *SHELXL97*.

## Supplementary Material

Crystal structure: contains datablock(s) global, I. DOI: 10.1107/S1600536813010787/tk5220sup1.cif

Structure factors: contains datablock(s) I. DOI: 10.1107/S1600536813010787/tk5220Isup2.hkl

Supplementary material file. DOI: 10.1107/S1600536813010787/tk5220Isup3.cml

Additional supplementary materials:  crystallographic information; 3D view; checkCIF report

## Figures and Tables

**Table 1:** Hydrogen-bond geometry (Å, °)

*D*—H⋯*A*	*D*—H	H⋯*A*	*D*⋯*A*	*D*—H⋯*A*
N1—H1*A*⋯O1^i^	0.89	1.89	2.709 (11)	152
N1—H1*B*⋯O2^ii^	0.89	1.88	2.760 (10)	168
N1—H1*C*⋯O2	0.89	1.90	2.752 (11)	159
O3—H3*A*⋯O2^iii^	0.82	1.88	2.674 (3)	162

Symmetry codes: (i) 
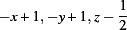
; (ii) 
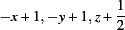
; (iii) 
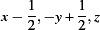
.

**Fig. 1.**
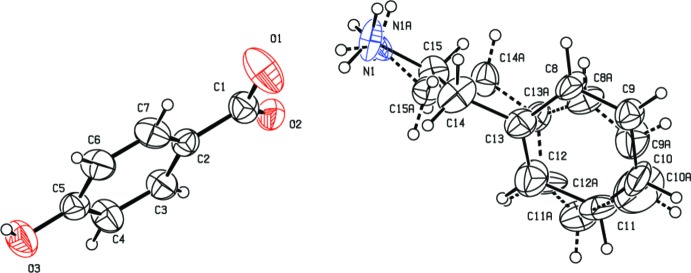
The molecular structure of (I), with atom labels and 30% probability displacement ellipsoids for non-H atoms.

**Fig. 2.**
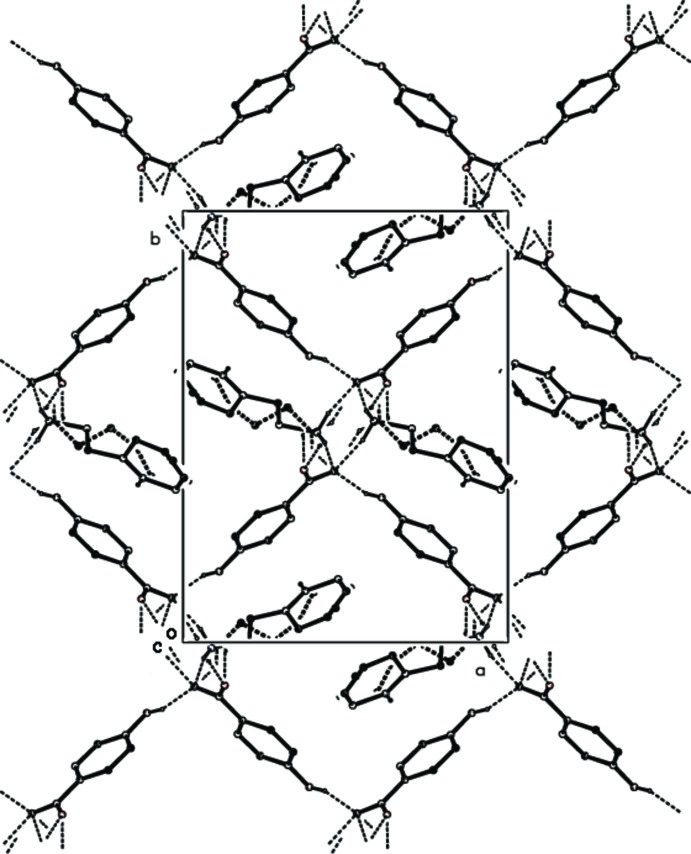
The packing of (I), viewed down c axis. Hydrogen bonds are shown as dashed lines. H atoms not involved in hydrogen bonding have been omitted.

## supplementary crystallographic information

### Comment

p-Hydroxybenzoate has been found to interact with different cations to form different crystal structures (Marsh *et al.*, 2001; Yang *et al.*, 2010). The asymmetric unit of the title compound, (I), Fig 1, contains a C_8_H_12_N^+^ cation and a C_7_H_5_O_3_^-^ anion. The bond lengths and angles of the anion are comparable with a recently reported structure (Sudhahar *et al.*, 2013). In the anion, the carboxylate group makes the dihedral angle of 4.19 (18) Å with the benzene ring. In the crystal structure, the anions and cations are connected by weak N—H···O and O—H···O interactions (Table 1 & Fig. 2) to form a three-dimensional network.

### Experimental

The title salt was synthesized from the starting materials of 2-phenylethylamine (1.26 g) and 4-hydroxybenzoic acid (1.38 g) taken in methanol:water mixed solvent system. Single crystals suitable for X-ray diffraction were grown by slow evaporation.

### Refinement

The H atoms were positioned geometrically with C—H = 0.93–0.97 Å O—H = 0.82 Å and N—H = 0.89 Å and allowed to ride on their parent atoms, with 1.2*U*_eq_(C) and *U*_iso_(H) = 1.5*U*_eq_(O,N). The cation is oriented over two sites with occupancies 0.565 (7) and 0.435 (7). During refinement, the same distance restraint (SADI 0.01) was applied to the following pairs of bonds: C13—C14 and C13A—C14A, C8—C13 and C8A—C13A, C14—C15 and C14A—C15A, and C15—N1 and C15A—N1A. The anisotropic displacement parameters of C11 and C12 were restrained with the DELU 0.01 instruction. Two reflections, i.e. (1 1 0) and (0 2 0), were omitted from the final cycles of refinement owing to poor agreement.

### Crystal data

**Table:**

C_8_H_12_N^+^·C_7_H_5_O_3_^−^	*F*(000) = 552
*M**_r_* = 259.30	*D*_x_ = 1.222 Mg m^−^^3^
Orthorhombic, *P**n**a*2_1_	Mo *K*α radiation, λ = 0.71073 Å
Hall symbol: P 2c -2n	Cell parameters from 2142 reflections
*a* = 13.0721 (12) Å	θ = 2.8–25.4°
*b* = 17.3426 (16) Å	µ = 0.09 mm^−^^1^
*c* = 6.2154 (6) Å	*T* = 295 K
*V* = 1409.1 (2) Å^3^	Block, colourless
*Z* = 4	0.36 × 0.30 × 0.24 mm

### Data collection

**Table:**

Bruker Kappa APEXII CCD diffractometer	2561 independent reflections
Radiation source: fine-focus sealed tube	1671 reflections with *I* > 2σ(*I*)
Graphite monochromator	*R*_int_ = 0.029
ω and φ scan	θ_max_ = 25.3°, θ_min_ = 2.8°
Absorption correction: multi-scan (*SADABS*; Sheldrick, 1996)	*h* = −13→15
*T*_min_ = 0.970, *T*_max_ = 0.980	*k* = −20→16
7327 measured reflections	*l* = −7→7

### Refinement

**Table:**

Refinement on *F*^2^	Secondary atom site location: difference Fourier map
Least-squares matrix: full	Hydrogen site location: inferred from neighbouring sites
*R*[*F*^2^ > 2σ(*F*^2^)] = 0.038	H-atom parameters constrained
*wR*(*F*^2^) = 0.106	*w* = 1/[σ^2^(*F*_o_^2^) + (0.0508*P*)^2^ + 0.0395*P*] where *P* = (*F*_o_^2^ + 2*F*_c_^2^)/3
*S* = 1.03	(Δ/σ)_max_ < 0.001
2561 reflections	Δρ_max_ = 0.12 e Å^−^^3^
254 parameters	Δρ_min_ = −0.13 e Å^−^^3^
6 restraints	Absolute structure: nd
Primary atom site location: structure-invariant direct methods	

### Special details

**Table:**

Geometry. All esds (except the esd in the dihedral angle between two l.s. planes) are estimated using the full covariance matrix. The cell esds are taken into account individually in the estimation of esds in distances, angles and torsion angles; correlations between esds in cell parameters are only used when they are defined by crystal symmetry. An approximate (isotropic) treatment of cell esds is used for estimating esds involving l.s. planes.
Refinement. Refinement of F^2^ against ALL reflections. The weighted R-factor wR and goodness of fit S are based on F^2^, conventional R-factors R are based on F, with F set to zero for negative F^2^. The threshold expression of F^2^ > 2sigma(F^2^) is used only for calculating R-factors(gt) etc. and is not relevant to the choice of reflections for refinement. R-factors based on F^2^ are statistically about twice as large as those based on F, and R- factors based on ALL data will be even larger.

### Fractional atomic coordinates and isotropic or equivalent isotropic displacement parameters (Å^2^)

**Table:**

	*x*	*y*	*z*	*U*_iso_*/*U*_eq_	Occ. (<1)
C1	0.3890 (2)	0.37442 (13)	0.6595 (4)	0.0748 (6)	
C2	0.31676 (16)	0.31851 (12)	0.5643 (4)	0.0604 (5)	
C3	0.33145 (19)	0.28736 (13)	0.3645 (4)	0.0733 (6)	
H3	0.3892	0.3009	0.2856	0.088*	
C4	0.26201 (19)	0.23621 (15)	0.2782 (4)	0.0811 (7)	
H4	0.2734	0.2158	0.1419	0.097*	
C5	0.17637 (18)	0.21509 (13)	0.3907 (4)	0.0694 (6)	
C6	0.16109 (19)	0.24534 (15)	0.5906 (4)	0.0816 (7)	
H6	0.1037	0.2311	0.6696	0.098*	
C7	0.2299 (2)	0.29658 (13)	0.6757 (4)	0.0803 (7)	
H7	0.2178	0.3171	0.8115	0.096*	
O2	0.46474 (11)	0.39682 (8)	0.5459 (3)	0.0800 (5)	
O3	0.11124 (14)	0.16400 (11)	0.2981 (3)	0.1025 (6)	
H3A	0.0636	0.1552	0.3803	0.154*	
O1	0.3714 (2)	0.40004 (14)	0.8429 (4)	0.1320 (9)	
C8	0.8544 (9)	0.4684 (9)	1.1607 (13)	0.082 (3)	0.565 (7)
H8	0.8219	0.5128	1.2091	0.099*	0.565 (7)
C9	0.9437 (12)	0.4412 (8)	1.2649 (19)	0.075 (3)	0.565 (7)
H9	0.9641	0.4615	1.3966	0.090*	0.565 (7)
C10	1.0025 (11)	0.3810 (10)	1.161 (4)	0.100 (5)	0.565 (7)
H10	1.0627	0.3644	1.2264	0.120*	0.565 (7)
C11	0.9770 (14)	0.3519 (11)	0.995 (3)	0.093 (5)	0.565 (7)
H11	1.0153	0.3116	0.9385	0.112*	0.565 (7)
C12	0.8778 (14)	0.3815 (13)	0.871 (2)	0.111 (6)	0.565 (7)
H12	0.8641	0.3676	0.7291	0.133*	0.565 (7)
C13	0.8154 (5)	0.4275 (4)	0.9833 (10)	0.0722 (17)	0.565 (7)
C14	0.7075 (5)	0.4460 (3)	0.9177 (10)	0.090 (2)	0.565 (7)
H14A	0.6711	0.3984	0.8889	0.108*	0.565 (7)
H14B	0.6732	0.4720	1.0355	0.108*	0.565 (7)
C15	0.7036 (4)	0.4958 (4)	0.7234 (8)	0.0794 (18)	0.565 (7)
H15A	0.7303	0.4689	0.5986	0.095*	0.565 (7)
H15B	0.7425	0.5428	0.7449	0.095*	0.565 (7)
N1	0.5880 (8)	0.5137 (6)	0.6959 (15)	0.084 (3)	0.565 (7)
H1A	0.5791	0.5442	0.5824	0.125*	0.565 (7)
H1B	0.5648	0.5372	0.8135	0.125*	0.565 (7)
H1C	0.5538	0.4698	0.6762	0.125*	0.565 (7)
C8A	0.8880 (10)	0.4599 (12)	1.148 (2)	0.098 (5)	0.435 (7)
H8A	0.8524	0.4913	1.2436	0.118*	0.435 (7)
C9A	0.9684 (16)	0.4220 (13)	1.212 (4)	0.115 (10)	0.435 (7)
H9A	0.9893	0.4314	1.3525	0.137*	0.435 (7)
C10A	1.018 (2)	0.378 (2)	1.116 (9)	0.22 (2)	0.435 (7)
H10A	1.0801	0.3586	1.1639	0.262*	0.435 (7)
C11A	0.967 (2)	0.3549 (19)	0.898 (4)	0.104 (7)	0.435 (7)
H11A	0.9950	0.3166	0.8119	0.125*	0.435 (7)
C12A	0.899 (2)	0.3841 (12)	0.852 (4)	0.108 (7)	0.435 (7)
H12A	0.8632	0.3613	0.7397	0.130*	0.435 (7)
C13A	0.8560 (6)	0.4526 (5)	0.9342 (13)	0.0609 (19)	0.435 (7)
C14A	0.7772 (4)	0.4966 (3)	0.8103 (10)	0.072 (2)	0.435 (7)
H14C	0.7680	0.5470	0.8755	0.086*	0.435 (7)
H14D	0.8012	0.5043	0.6642	0.086*	0.435 (7)
C15A	0.6773 (5)	0.4558 (4)	0.8050 (15)	0.0655 (19)	0.435 (7)
H15C	0.6526	0.4460	0.9497	0.079*	0.435 (7)
H15D	0.6834	0.4070	0.7296	0.079*	0.435 (7)
N1A	0.6043 (9)	0.5113 (6)	0.6832 (19)	0.072 (4)	0.435 (7)
H1E	0.5424	0.4902	0.6743	0.108*	0.435 (7)
H1F	0.6284	0.5198	0.5514	0.108*	0.435 (7)
H1D	0.6002	0.5558	0.7541	0.108*	0.435 (7)

### Atomic displacement parameters (Å^2^)

**Table:**

	*U*^11^	*U*^22^	*U*^33^	*U*^12^	*U*^13^	*U*^23^
C1	0.0827 (17)	0.0731 (14)	0.0685 (16)	−0.0049 (14)	−0.0114 (14)	0.0053 (14)
C2	0.0601 (13)	0.0590 (12)	0.0620 (13)	0.0042 (11)	−0.0004 (11)	0.0058 (11)
C3	0.0682 (15)	0.0760 (15)	0.0757 (16)	−0.0053 (13)	0.0144 (12)	−0.0077 (13)
C4	0.0795 (17)	0.0930 (18)	0.0707 (16)	−0.0163 (15)	0.0152 (13)	−0.0154 (14)
C5	0.0637 (14)	0.0664 (14)	0.0781 (16)	0.0031 (12)	0.0045 (12)	−0.0060 (12)
C6	0.0730 (15)	0.0774 (15)	0.0944 (19)	−0.0112 (14)	0.0266 (14)	−0.0100 (15)
C7	0.0987 (18)	0.0776 (15)	0.0645 (14)	−0.0058 (15)	0.0200 (14)	−0.0092 (13)
O2	0.0606 (9)	0.0745 (9)	0.1050 (12)	−0.0018 (8)	−0.0089 (10)	−0.0008 (10)
O3	0.0831 (11)	0.1104 (14)	0.1141 (16)	−0.0279 (11)	0.0056 (10)	−0.0291 (12)
O1	0.173 (2)	0.148 (2)	0.0746 (13)	−0.0583 (18)	−0.0001 (14)	−0.0254 (13)
C8	0.071 (7)	0.110 (5)	0.065 (4)	0.046 (5)	−0.002 (4)	−0.018 (3)
C9	0.072 (7)	0.088 (4)	0.065 (5)	0.008 (5)	−0.008 (5)	0.004 (4)
C10	0.039 (5)	0.102 (8)	0.157 (13)	0.012 (5)	−0.020 (6)	0.030 (10)
C11	0.076 (7)	0.071 (6)	0.133 (15)	0.021 (6)	0.040 (9)	0.013 (11)
C12	0.103 (9)	0.133 (11)	0.096 (9)	0.015 (7)	−0.010 (9)	−0.021 (7)
C13	0.067 (4)	0.077 (4)	0.072 (4)	0.009 (3)	0.018 (3)	−0.006 (3)
C14	0.088 (5)	0.111 (4)	0.072 (4)	0.023 (4)	0.023 (3)	0.026 (3)
C15	0.070 (5)	0.101 (4)	0.067 (3)	−0.009 (3)	0.006 (3)	−0.004 (3)
N1	0.047 (3)	0.131 (7)	0.073 (5)	−0.008 (3)	−0.014 (3)	0.031 (4)
C8A	0.060 (9)	0.113 (9)	0.122 (10)	0.036 (8)	0.017 (7)	−0.041 (6)
C9A	0.070 (13)	0.18 (2)	0.097 (12)	0.009 (13)	0.010 (8)	−0.001 (13)
C10A	0.15 (2)	0.19 (2)	0.32 (4)	0.011 (17)	−0.01 (3)	0.00 (3)
C11A	0.085 (9)	0.096 (12)	0.131 (16)	0.024 (7)	0.039 (11)	−0.024 (12)
C12A	0.133 (13)	0.075 (8)	0.116 (11)	0.037 (8)	0.082 (9)	−0.018 (6)
C13A	0.042 (4)	0.072 (5)	0.068 (5)	−0.002 (4)	0.007 (3)	0.003 (3)
C14A	0.058 (4)	0.076 (4)	0.080 (4)	−0.014 (3)	0.000 (3)	0.012 (3)
C15A	0.065 (4)	0.053 (3)	0.079 (5)	−0.008 (3)	−0.005 (4)	0.017 (4)
N1A	0.062 (7)	0.084 (6)	0.069 (6)	−0.017 (4)	0.012 (4)	−0.042 (5)

### Geometric parameters (Å, º)

**Table:**

C1—O1	1.245 (3)	C14—H14B	0.9700
C1—O2	1.277 (3)	C15—N1	1.551 (8)
C1—C2	1.477 (3)	C15—H15A	0.9700
C2—C3	1.368 (3)	C15—H15B	0.9700
C2—C7	1.383 (3)	N1—H1A	0.8900
C3—C4	1.378 (3)	N1—H1B	0.8900
C3—H3	0.9300	N1—H1C	0.8900
C4—C5	1.370 (3)	C8A—C9A	1.30 (3)
C4—H4	0.9300	C8A—C13A	1.401 (11)
C5—O3	1.357 (3)	C8A—H8A	0.9300
C5—C6	1.363 (3)	C9A—C10A	1.16 (5)
C6—C7	1.370 (3)	C9A—H9A	0.9300
C6—H6	0.9300	C10A—C11A	1.56 (6)
C7—H7	0.9300	C10A—H10A	0.9300
O3—H3A	0.8200	C11A—C12A	1.06 (4)
C8—C13	1.407 (9)	C11A—H11A	0.9300
C8—C9	1.415 (19)	C12A—C13A	1.41 (2)
C8—H8	0.9300	C12A—H12A	0.9300
C9—C10	1.45 (3)	C13A—C14A	1.495 (7)
C9—H9	0.9300	C14A—C15A	1.486 (7)
C10—C11	1.20 (3)	C14A—H14C	0.9700
C10—H10	0.9300	C14A—H14D	0.9700
C11—C12	1.60 (2)	C15A—N1A	1.554 (8)
C11—H11	0.9300	C15A—H15C	0.9700
C12—C13	1.34 (2)	C15A—H15D	0.9700
C12—H12	0.9300	N1A—H1E	0.8900
C13—C14	1.503 (8)	N1A—H1F	0.8900
C14—C15	1.485 (6)	N1A—H1D	0.8900
C14—H14A	0.9700		
			
O1—C1—O2	122.8 (2)	C14—C15—H15A	111.0
O1—C1—C2	118.9 (2)	N1—C15—H15A	111.0
O2—C1—C2	118.3 (2)	C14—C15—H15B	111.0
C3—C2—C7	117.4 (2)	N1—C15—H15B	111.0
C3—C2—C1	122.2 (2)	H15A—C15—H15B	109.0
C7—C2—C1	120.3 (2)	C15—N1—H1A	109.5
C2—C3—C4	121.0 (2)	C15—N1—H1B	109.5
C2—C3—H3	119.5	H1A—N1—H1B	109.5
C4—C3—H3	119.5	C15—N1—H1C	109.5
C5—C4—C3	120.8 (2)	H1A—N1—H1C	109.5
C5—C4—H4	119.6	H1B—N1—H1C	109.5
C3—C4—H4	119.6	C9A—C8A—C13A	119.0 (11)
O3—C5—C6	123.1 (2)	C9A—C8A—H8A	120.5
O3—C5—C4	118.1 (2)	C13A—C8A—H8A	120.5
C6—C5—C4	118.8 (2)	C10A—C9A—C8A	129 (3)
C5—C6—C7	120.3 (2)	C10A—C9A—H9A	115.7
C5—C6—H6	119.8	C8A—C9A—H9A	115.7
C7—C6—H6	119.8	C9A—C10A—C11A	112 (3)
C6—C7—C2	121.6 (2)	C9A—C10A—H10A	124.0
C6—C7—H7	119.2	C11A—C10A—H10A	124.0
C2—C7—H7	119.2	C12A—C11A—C10A	118 (3)
C5—O3—H3A	109.5	C12A—C11A—H11A	121.0
C13—C8—C9	119.3 (11)	C10A—C11A—H11A	121.0
C13—C8—H8	120.4	C11A—C12A—C13A	130 (3)
C9—C8—H8	120.4	C11A—C12A—H12A	115.2
C8—C9—C10	118.4 (13)	C13A—C12A—H12A	115.2
C8—C9—H9	120.8	C8A—C13A—C12A	107.5 (13)
C10—C9—H9	120.8	C8A—C13A—C14A	130.5 (9)
C11—C10—C9	122.6 (13)	C12A—C13A—C14A	121.2 (12)
C11—C10—H10	118.7	C15A—C14A—C13A	111.9 (5)
C9—C10—H10	118.7	C15A—C14A—H14C	109.2
C10—C11—C12	120.7 (16)	C13A—C14A—H14C	109.2
C10—C11—H11	119.7	C15A—C14A—H14D	109.2
C12—C11—H11	119.7	C13A—C14A—H14D	109.2
C13—C12—C11	115.7 (11)	H14C—C14A—H14D	107.9
C13—C12—H12	122.2	C14A—C15A—N1A	104.8 (8)
C11—C12—H12	122.2	C14A—C15A—H15C	110.8
C12—C13—C8	119.3 (9)	N1A—C15A—H15C	110.8
C12—C13—C14	123.9 (8)	C14A—C15A—H15D	110.8
C8—C13—C14	116.4 (7)	N1A—C15A—H15D	110.8
C15—C14—C13	112.2 (5)	H15C—C15A—H15D	108.9
C15—C14—H14A	109.2	C15A—N1A—H1E	109.5
C13—C14—H14A	109.2	C15A—N1A—H1F	109.5
C15—C14—H14B	109.2	H1E—N1A—H1F	109.5
C13—C14—H14B	109.2	C15A—N1A—H1D	109.5
H14A—C14—H14B	107.9	H1E—N1A—H1D	109.5
C14—C15—N1	103.8 (6)	H1F—N1A—H1D	109.5
			
O1—C1—C2—C3	−179.4 (3)	C11—C12—C13—C8	23 (2)
O2—C1—C2—C3	3.5 (3)	C11—C12—C13—C14	−164.8 (12)
O1—C1—C2—C7	1.5 (3)	C9—C8—C13—C12	−22.7 (19)
O2—C1—C2—C7	−175.6 (2)	C9—C8—C13—C14	164.2 (10)
C7—C2—C3—C4	0.1 (3)	C12—C13—C14—C15	−69.6 (14)
C1—C2—C3—C4	−179.0 (2)	C8—C13—C14—C15	103.1 (10)
C2—C3—C4—C5	−0.1 (4)	C13—C14—C15—N1	−175.2 (6)
C3—C4—C5—O3	−179.5 (2)	C13A—C8A—C9A—C10A	−4 (4)
C3—C4—C5—C6	−0.3 (4)	C8A—C9A—C10A—C11A	−10 (5)
O3—C5—C6—C7	179.8 (2)	C9A—C10A—C11A—C12A	6 (5)
C4—C5—C6—C7	0.7 (4)	C10A—C11A—C12A—C13A	14 (5)
C5—C6—C7—C2	−0.8 (4)	C9A—C8A—C13A—C12A	20 (3)
C3—C2—C7—C6	0.4 (3)	C9A—C8A—C13A—C14A	−170.4 (16)
C1—C2—C7—C6	179.4 (2)	C11A—C12A—C13A—C8A	−26 (4)
C13—C8—C9—C10	11.5 (18)	C11A—C12A—C13A—C14A	163 (3)
C8—C9—C10—C11	−2 (2)	C8A—C13A—C14A—C15A	−96.9 (15)
C9—C10—C11—C12	3 (2)	C12A—C13A—C14A—C15A	71.6 (15)
C10—C11—C12—C13	−14 (3)	C13A—C14A—C15A—N1A	176.8 (7)

### Hydrogen-bond geometry (Å, º)

**Table:**

*D*—H···*A*	*D*—H	H···*A*	*D*···*A*	*D*—H···*A*
N1—H1*A*···O1^i^	0.89	1.89	2.709 (11)	152
N1—H1*B*···O2^ii^	0.89	1.88	2.760 (10)	168
N1—H1*C*···O2	0.89	1.90	2.752 (11)	159
O3—H3*A*···O2^iii^	0.82	1.88	2.674 (3)	162

Symmetry codes: (i) −*x*+1, −*y*+1, *z*−1/2; (ii) −*x*+1, −*y*+1, *z*+1/2; (iii) *x*−1/2, −*y*+1/2, *z*.
